# Respiratory support of adults in the emergency department: A protocol for a prospective, observational, multicenter point prevalence study

**DOI:** 10.1002/hsr2.966

**Published:** 2022-11-30

**Authors:** Jane O'Donnell, Alison Pirret, Karen Hoare, Elissa McDonald

**Affiliations:** ^1^ School of Nursing, College of Health, Massey University Albany Campus Auckland New Zealand

**Keywords:** COVID 19, emergency, nasal high flow, point prevalence, respiratory support

## Abstract

**Background and Aims:**

Providing respiratory support (RS) to patients may improve their oxygenation and ventilation, reducing the work of breathing. Emergency department (ED) patients often need RS; COVID‐19 has heightened this need. Patients receiving RS may need escalation of their treatment; hence, studies considering the prevalence of escalation are warranted.

**Method:**

This is a protocol for a prospective, observational, multicenter point prevalence study (PPS). Researchers will collect data over 2 days. All participants are adult ED patients needing RS. The setting is four EDs in New Zealand. The primary research question asks, “Which patients receiving RS require escalation of therapy in the ED?” For example, transitioning from conventional oxygen therapy (COT) to intubation is deemed an escalation of therapy. A sample size of 80 participants is required to resolve the primary research question. Secondary research questions: (1) Which patients receive nasal high flow (NHF) in the ED? (2) How is NHF therapy delivered in the ED? (3) What are the effects of NHF therapy on physiological and patient‐centered outcomes? Research Electronic Data Capture (REDCap) will be used for data organization. Data will be imported for analysis from REDCap to IBM SPSS software (Statistics for Windows, Version 27.0). Data reporting on the primary outcome shall be considered by analysis of variance, regression modeling, and determination of two treatment effects: Odds Ratio and Number Needed to Treat. Statistical significance for inferential statistics shall use a two‐sided *α* with *p*‐values fixed at ≤0.05 level of significance and 95% confidence intervals. This protocol has ethical approval from Massey University, New Zealand.

**Conclusion:**

This novel PPS may reduce the evidence and clinical practice gap on RS delivery and ED patient outcomes, as evidenced by the emergence of COVID‐19.

## 1 INTRODUCTION

### 1.1 Background

#### 1.1.1 Problem statement

Many emergency department (ED) patients require respiratory support (RS) to optimize their oxygenation and limit the likelihood of requiring escalation of their care. However, the effects of escalating care include an increase in morbidity and mortality risk.

The limited availability of evidence focusing on RS delivery in the ED justifies an investigation. This protocol has been developed to facilitate an observational multicentre point prevalence study (PPS).

Patients reporting to the ED often need RS.^1^ These RS therapies can decrease the work of breathing.^2^ Many of these presenting patients also have chronic respiratory disease^3^; however, acute ED patients may also need RS, such as patients with trauma, infection, or those experiencing side effects of other interventions.^4^

RS is an umbrella term for a collection of therapies. The collection includes conventional oxygen therapy (COT), also referred to as standard oxygen therapy (using traditional masks and cannulae); nasal high flow (NHF); noninvasive (NIV); and invasive ventilation (INV). Clinicians provide RS therapies either in an invasive or NIV manner. Invasive RS involves intubation and mechanical ventilation. NIV RS includes COT, NHF, and two forms of NIV: bilevel positive airway pressure and continuous positive airway pressure.

Several of these RS therapies are in use in the ED. The most novel of these RS therapies is NHF, the focus of this study.^5^

NHF RS provides high flows of warmed, humidified gas (at up to 60 L/min).^5^ Gas temperature (31–37°C), gas flow (2–60 L/min), and the inspired oxygen fraction (FiO_2_) (21%–100%) are independently titrated. The delivery of gases to spontaneously breathing patients is via specialized nasal cannulae.^6^ Despite the lack of reliable and valid evidence, the use of this RS therapy in the ED is widespread.^7^

Clinicians accept that improved patient outcomes rely on evidence‐based care.^8^ However, since 2000, the adult NHF evidence and NHF use have accelerated disproportionally. To date, PubMed has indexed more than 3900 NHF publications. Yet, most of these NHF publications are case reports or observational studies with an intensive care focus.^7^ Hence, ED clinicians have limited NHF‐controlled studies to inform their clinical practice.^9^ The limited availability of reliable and valid evidence provides the rationale for this study.

The first NHF‐controlled study reported that NHF could reduce respiratory rates of ED patients (21–28 breaths/min) (*p* < 0.001).^10^ Two later studies confirmed Lenglet's findings suggesting the efficacy and feasibility of NHF in the ED.^11^, ^12^ These controlled studies have motivated completing a New Zealand randomized controlled trial (RCT).

The New Zealand study was the first large ED RCT that compared NHF to COT.^13^ The primary study outcome was a need for escalation to NIV or INV. The study identified no difference in the need for escalation between NHF (3.6%, 95% CI: 1.5%–7.9%) and COT (7.3%, 95% CI: 3.8%–13%) (*p* = 0.16).

Nevertheless, this study was underpowered, with 40% of the target sample recruited. Even so, the researchers concluded that NHF did not reduce the need for escalation to NIV or INV compared to COT.

Researchers involved in this initial RCT have subsequently completed a multicentre ED PPS based in the Asia‐Pacific region. The PPS described the epidemiology of 3044 patients experiencing dyspnea.^3^

The authors of this PPS confirmed that ED patients often present with dyspnea in this region. However, despite these efforts, large gaps remain in the evidence describing RS delivery and patient outcomes.

Despite the lack of high‐quality primary ED evidence, several NHF systematic reviews are now published.^1^, ^14^, ^15^, ^16^ But, the lack of valid and reliable evidence has then impacted the validity and reliability of these reviews, limiting their applicability in clinical practice and research.^14^ This proposed PPS is novel and may reduce the evidence and clinical practice gap regarding RS delivery and ED patient outcomes, as evidenced by the emergence of COVID‐19.^17^

### 1.2 Study objectives and aims

All research should look to inform clinicians and improve patient outcomes. To achieve this, researchers must explore outcomes of clinical significance.^18^ This proposed research aims to examine the nature of RS delivered in the ED and its influence on the escalation of therapy in the ED. As such, the outcomes of this study will have clinical significance.

The primary research question is:

- Which patients receiving RS require escalation of therapy in the ED?

Also, three secondary research questions focus on the RS NHF:

- Which patients receive NHF in the ED?
- How is NHF therapy delivered in the ED?
- What are the effects of NHF therapy on physiological parameters and escalation of therapy?

## 2 METHODS

### 2.1 Study design

This study design is a prospective, observational multicenter, PPS. Of note, the researchers acknowledge that the RCT is the gold standard when considering the efficacy of any therapy, including NHF therapy.^19^ But as NHF therapy has become ubiquitous, undermining the ethics of conducting RCTs involving NHF,^20^ the researchers are convinced that an observational research design selection is now ethically validated.^21^ Furthermore, this observational PPS design enables rapid and reliable data collection from a large ED patient cohort.^22^ Finally, this protocol describes the planned observational multicenter PPS.

### 2.2 Study setting

This study is set in New Zealand EDs. The researchers are aware of the importance of study selection. Poor site selection can undermine the reliability of a study and even threaten its completion.^23^ Therefore, the researchers selected four New Zealand EDs. Two participating sites are tertiary referral EDs in a large metropolitan city, and two are regional EDs in smaller cities. Data collection is due to be completed by December 2022.

### 2.3 Study conditions

This is an observational multicenter PPS. There are no study controls. Experimental conditions are not applied in this study.

### 2.4 Study participants

Establishing an appropriate target population must align with the study outcomes under consideration.^18^ Therefore, the target population is ED patients 18 years or older who need RS.

These patients may have respiratory insufficiencies or have side effects related to other interventions.^4^ This population aligns with the study outcomes.

#### 2.4.1 Study participant recruitment

The methods used in this study mean that participants do not need active management postinclusion, unlike in the RCT.^21^ Instead, the data collectors shall use predetermined inclusion criteria to screen potential participants for inclusion (Table 1). The data collectors shall document the screening process in a screening log. Researchers shall collect data for every ED patient meeting the criteria for inclusion during two prespecified 12‐h data collection periods, each separated by 4 weeks (Tables 2 and 3).

**Table 1:** Study screening criteria

Inclusion criterion	Exclusion criterion
ED patients aged ≥18 years needing any form or combination of RS (via COT, NIV, NHF, BiPaP, CPAP, and or INV) during one of the two study intervals	ED patients aged ≥18 years not needing any form or combination of RS (via COT, NIV, NHF, BiPaP, CPAP, and or INV) during one of the two study intervals

Abbreviations: BiPaP, bilevel positive airway pressure; COT, conventional oxygen therapy; CPAP, continuous positive airway pressure; ED, emergency department; INV, invasive ventilation; NHF, nasal high flow; NIV, noninvasive.

**Table 2:** Study data

Categories of data captured in the Screening Log	Categories of outcome variable data captured eCRF Part A	Categories of outcome variable data captured eCRF Part B
Screening log number	Comorbidities	NHF‐related adverse events in ED
NHI^a^	Medications (usual)	Duration of NHF in ED
Name (surname first)	Mode of arrival to ED	Requirement of escalation of RS in ED
Age YEARS	Prehospital treatment by ambulance	Rationale for escalation of RS
Gender M, F, O	Initial treatment in the ED	Type of escalation of RS
Date screened for inclusion DD/MM/YY.	Initial ED assessment	NHF therapy data (physiological outcomes and delivery of therapy)
Time screened for inclusion. 24 h. HH:MM	Investigations in ED	NHF‐related adverse events in ED
Included Y or N	Treatment in the ED after 1st formal assessment	
Reason excluded. A or B	ED diagnosis	
ED data collection complete Y/N	Cause of RS requirement in ED	
HOSP FU data are complete Y/N	RS on ED discharge	
Participating hospital. (A, B, C, or D)	ED discharge destination	
Ethnicity	Final hospital discharge date	
Reason excluded. A or B	Final hospital discharge diagnosis	
ED data collection complete Y/N		
HOSP FU data are complete Y/N		
Participating hospital. (A, B, C, or D)		
COVID‐19 status		
COVID‐19 vaccination status		

Abbreviations: eCRF, electronic case report form; ED, emergency department; NHF, nasal high flow; RS, respiratory support.

^a^ New Zealand National Health Index Number.

**Table 3:** Research questions, outcome variables, data type, and data collection time points

	Outcome variables	Data type	Data collection timepoint
Primary question			
Which patients receiving RS require escalation of therapy in the ED?	Requirement for escalation of care	Dichotomous	While in ED
	ED diagnostic category	Categorical	While in ED
Secondary questions			
Which patients receive NHF in the ED?	ED diagnostic category	Categorical	While in ED
	Duration of RS in hours	Continuous	While in ED
	Type of RS applied in ED	Categorical	While in ED
How is NHF therapy delivered in the ED?	Mortality while in hospital (this admission)	Dichotomous	During this hospitalization
	Mortality while in ED	Dichotomous	While in ED
	Adverse event^a^ in ED	Categorical	While in ED
	Hospital length of stay in days	Continuous	During this hospitalization
	ED length of stay in hours	Continuous	While in ED
	ED discharge destination	Categorical	While in ED
	Physiological effect: SpO_2_	Continuous	While in ED
	Physiological effect: respiratory rate	Continuous	While in ED
	Physiological effect: HR	Continuous	While in ED
What are the effects seen with NHF therapy on physiological and patient‐centered outcomes?	Duration of NHF in hours	Continuous	While in ED
	NHF flow rate L/min	Continuous	While in ED
	NHF FiO_2_%	Continuous	While in ED

Abbreviations: ED, emergency department; FiO_2_, fraction of inspired oxygen; HR, heart rate; NHF, nasal high flow; RS, respiratory support; SpO_2_, oxygen saturation %.

^a^ Adverse event: (i) accidental harm or complication, which (ii) caused a death, disability, lengthening of the hospitalization, or lengthening of the usual signs and symptoms of a disease or condition; and (iii) attributed to the care provided rather than the patient's underlying disease.^24^

#### 2.4.2 Protection of study participants

All participants in clinical research need to be protected. The International Council for Harmonization (ICH) guideline^25^ “Technical Requirements for Pharmaceuticals for Human Use” proposes that the risks and benefits of a study must be evaluated and justified before a study begins. The researchers have assessed the risks and benefits of this study. This PPS observes ED standard clinical practice. The study has no experimental intervention or additional data collection. Therefore, the researchers have determined that this study poses no additional risk or benefit to the participants. As this study observes standard practice, researchers or data collectors shall not seek participant informed consent. The data collected shall be deidentified, kept confidential, and not used in ancillary studies.

The proposed study has gained full ethical approval (reference number: NOR 22/18) from the Massey University New Zealand Ethics Committee.^26^ In addition, the researchers shall procure the appropriate hospital site‐specific locality approvals.

The researchers will uphold transparency. The research protocol is available on a clinical trial registry (ACTRN12621001167853p U1111‐1262‐086). Furthermore, the researchers shall present the findings of this study at national and international conferences. Also, the researchers shall publish the study findings in peer‐reviewed quartile one journals. The researchers listed in this protocol are responsible and eligible for authorship. Finally, the researchers shall report the study findings to hospital representatives.

### 2.5 Study procedures

Appraisals of observational studies often question their validity and reliability based on the usual lack of available detail regarding study procedures.^27^

In contrast, peer review and publication of this study protocol shall provide the details for the proposed PPS procedures. This peer‐reviewed published protocol will support the appraisal of the PPS when completed.

**Figure 1:**
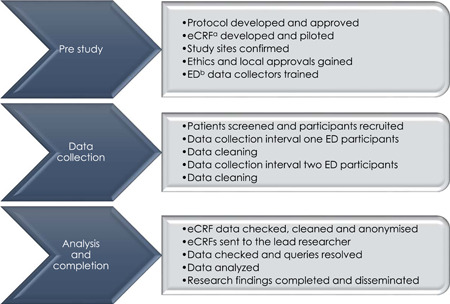
Study procedural phases and steps. ^a^Electronic case report form; ^b^emergency department.

This study has three procedural phases. The first phase is prestudy, the second is data collection, and the final phase is analysis and completion. Each study phase has five procedural steps (Figure 1). The researchers shall complete the study phases and steps in sequence. The guidelines provided by Assel et al.^28^ and Strengthening the Reporting of Observational Studies in Epidemiology (STROBE)^27^ were both used by the researchers while developing this protocol.

#### 2.5.1 Study data collection

Inaccurate data collection may invalidate the findings of a study.^29^ The researchers are responsible for all aspects of data collection and management. To ensure the rigor of the data collection, dedicated data collectors shall be trained and resourced at each site.

During each data collection period, data collectors must screen and include study participants using the inclusion criteria (Table 1). Also, data collectors shall allocate a unique study number (USN) to each included study participant.

These USNs shall help protect the participants' anonymity. In addition, data collectors shall document the screening process and the allocated USN in an electronic screening log. Also, data collectors shall complete one (Parts A and B) electronic case report form (eCRF) per study participant. The eCRF is purpose‐built and piloted to capture all study data (Table 2).

The researchers have no plans for external data monitoring or interim analyses. Thus, guidelines for the early termination of the study due to safety concerns are not required for this study.

#### 2.5.2 Handling of missing study data

Missing study data can undermine a study's findings.^18^ The PPS design maximizes an opportunity for data collection while reducing the risk of missing data. The descriptive analysis shall also identify any missing data. The strategy for dealing with missing data will be to use a process of regression imputation.^30^ This strategy involves the replacement of data that is missing with estimated values. This strategy shall ensure the maintenance of the study sample.^31^

#### 2.5.3 Study data security procedures

The researchers and data collectors must maintain the security of all study data.^25^

The security measures meet the requirements of the approving ethics committee and the participating hospitals. The secure internet‐based application Research Electronic Data Capture (REDCap; project-redcap.org) shall host the screening logs and the eCRFs. Researchers shall have access to the eCRFs data alone. All electronic devices that collect and send data will be password protected and shall have additional firewall security.

### 2.6 Study variables

Establishing the appropriate outcome variables requires alignment with the study objectives and questions.^18^ The study considers one primary and three secondary questions. The study outcome variables support the four research questions.^32^

Each research question involves composite variables comprising several normally correlated outcome variables.^32^ Table 3 positions each research question with the respective outcome variables, data types, and data collection time points.

**Figure 2:**
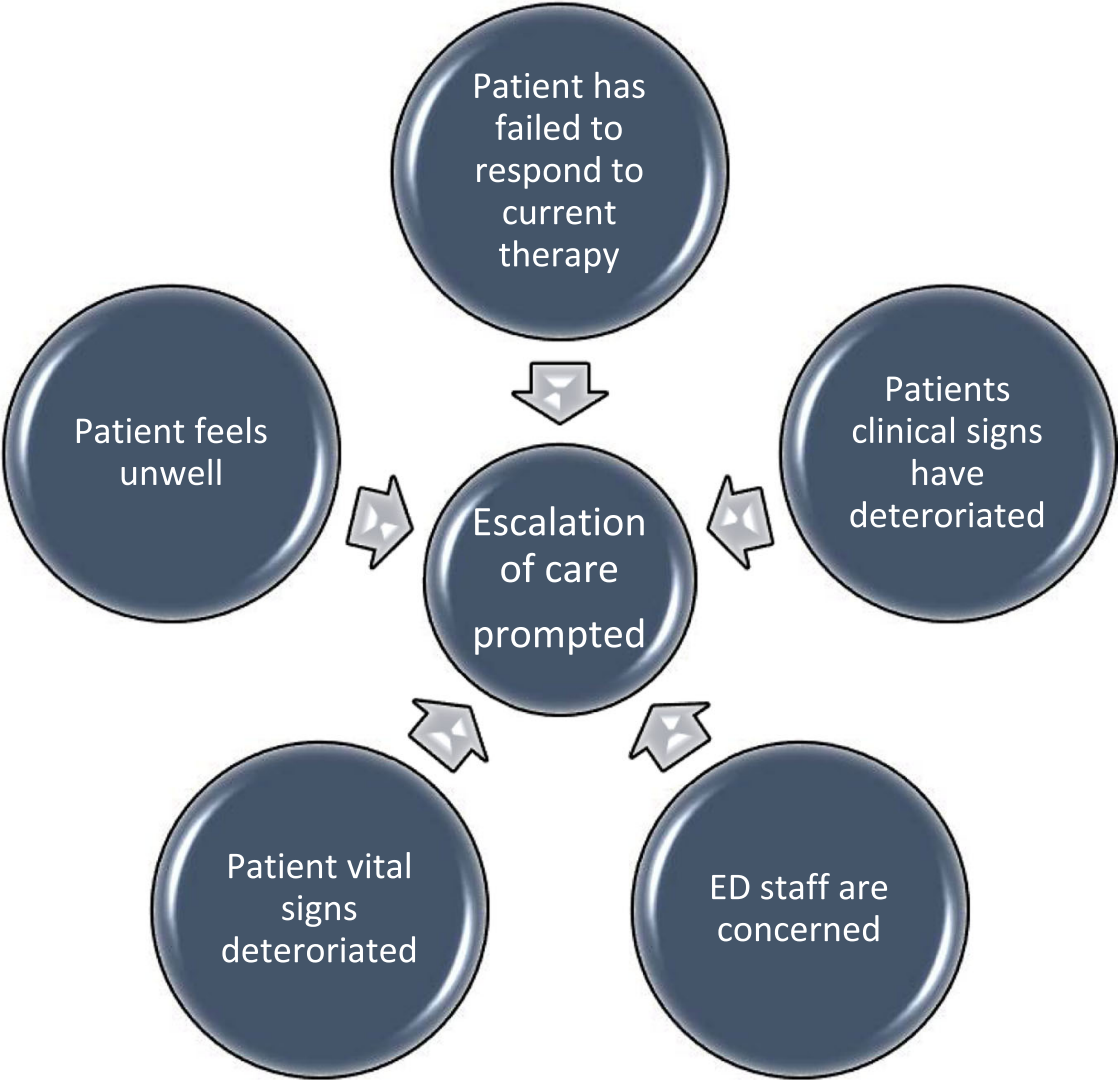
Triggers for escalation of care in the emergency department

The primary research question is, “Which patients receiving RS require escalation of therapy in the ED?” Often in ED, one or more escalation triggers indicate a need for escalation of patient care. So, the primary question considers whether a patient meets the threshold for escalation by meeting one or more of the nominated triggers for escalation (Figure 2).

The three secondary questions lend supporting evidence to the primary question. The three questions are:

(1) Which patients receive NHF in the ED?
(2) How is NHF therapy delivered in the ED
(3) What are the effects of NHF therapy on physiological and patient‐centered outcomes?

### 2.7 Study sample size

Study design involves establishing a representative sample of the study population. Without this, no reliable study conclusions can be made. Modeling can determine the appropriate sample size.^33^

This study involves ED patients receiving RS as a representative sample from the entire ED population presenting during the two data collection periods. A 95% confidence interval (CI) with a 5% margin of error (*α* = 0.05) will be applied. Sample size modeling for this study provided a sample size of 80 participants (Table 4). This modeling computes the minimum number of necessary samples to meet the desired statistical constraints. It incorporates the mean incidence of escalation to INV or NIV of 5.45%, as reported by Jones.^13^ Any reduction in this INV or NIV incidence was deemed clinically significant.

**Table 4:** Sample size model

	Model
Incidence of escalation to INV or NIV in the population	5.45%
Alpha	0.05
Power	80%
Sample size target	80

Abbreviations: INV, invasive ventilation; NIV, noninvasive.

### 2.8 Study analytical methods

Following data collection, the researchers must review the eCRFs for data discrepancies. Any discrepancies must be resolved in REDCap before data exportation to IBM SPSS (Statistics for Windows, Version 27.0) for statistical analysis.

The researchers shall perform two‐phased analyzes of the study data. Phase one shall involve a descriptive analysis of the data. This description shall include a narrative overview and analysis using central tendency and percentage measures. Normally distributed continuous data shall be described with means and standard deviations, and nonnormally distributed continuous data shall be described with medians and interquartile ranges. In addition, both categorical and dichotomous data will be reported as frequencies and percentages.

Completion of inferential analysis of data shall occur in the second phase. The inferential analysis plan was developed for each data type (categorical, continuous, and dichotomous).^33^ The inferential study analysis will be completed using IBM SPSS software (Statistics for Windows, Version 27.0).

#### 2.8.1 Study statistical methods

Selecting the most appropriate statistical test to analyze study data is vital.^35^ Therefore, the researchers consulted with a statistician when developing this study protocol. The statistical significance for all inferential analyzes shall use a two‐sided *α* with *p*‐values fixed at a ≤0.05 level of significance and CI set at 95%. Also, the Bonferroni method shall adjust the overall significance level for the three secondary outcomes.^36^

The inferential analysis methods shall include analysis of variance (ANOVA), regression modeling, and determination of treatment effects. First, the one‐way ANOVA shall compare primary outcome data for escalation between the four RS therapies.

Then regression modeling will estimate the relationships between the dependent and selected independent variables. Finally, the dependent variable for the logistic regression modeling shall be a requirement for escalation while in the ED, and the covariates, such as age, shall be the independent variables.

The dichotomous and categorical outcome data analysis shall be done with logistic regression. In addition, continuous data (such as ED length of stay) shall be analyzed using linear regression. These analyzes shall test the suitability and fitness of the models.

If available, survival using the time‐to‐event data (i.e., mortality) shall be analyzed using the Cox proportional hazards regression model. Finally, two treatment effects, Odds Ratio and Number Needed to Treat, shall be reported for the primary outcome. These treatment effects may describe the respective causal effect of the RS therapies.

## 3 DISCUSSION

Providing high‐quality ED care is essential to lowering the global health burden and better safeguarding public health in emergencies and epidemics. For example, the global COVID‐19 pandemic drove a significant influx of seriously ill patients to the ED, quickly overwhelming them. NIV RS has been commonplace during the pandemic because of the scarcity of ventilators, the high mortality of intubated patients, and the high infection risk among healthcare professionals involved in intubation. In addition to the unique physiological effects of NHF, individuals with COVID‐19 could comfortably receive high fractions of humidified FiO_2_.^17^ However, the evidence reporting on the use of NHF in ED patients is still yet to fully emerge. Of note, there are particular methodological and practical difficulties when doing research in the ED setting. Therefore, as a first step, research priorities for ED research must be established using epidemiological studies such as this.

## 4 CONCLUSION

This is a description of a protocol for a prospective multicenter observational PPS. This study is to be conducted in four New Zealand EDs. The protocol has been confirmed, the trial registered, and ethical approval obtained. The study and its reporting reflect shall the guidance and the standards as described by STROBE and ICH.^25^, ^27^

The delivery of RS may improve patient outcomes in the ED. This observational research may contribute to the evidence required to help better those who need it and those who provide RS in this setting.

All authors have read and approved the final version of the manuscript. Jane O'Donnell has full access to this proposed study's data. She takes complete responsibility for the data's integrity and the data analysis's accuracy. The data contributing to the final study's findings may be available from Jane O'Donnell following reasonable request.

## AUTHOR CONTRIBUTIONS

**Jane O'Donnell**: Conceptualization; methodology; project administration; resources; visualization; writing – original draft; writing – review & editing. **Alison Pirret**: Methodology; supervision; writing – review & editing. **Karen Hoare**: Supervision; writing – review & editing. **Elissa McDonald**: Supervision; writing – review & editing.

## CONFLICT OF INTEREST

The authors declare no conflict of interest.

## TRANSPARENCY STATEMENT

The lead author Jane O'Donnell affirms that this manuscript is an honest, accurate, and transparent account of the study being reported; that no important aspects of the study have been omitted; and that any discrepancies from the study as planned (and, if relevant, registered) have been explained.

## Data Availability

This manuscript describes a protocol for a study yet to be completed. As such, there are no data yet generated. When completed, data supporting the study's findings may be made available on request from the corresponding author. The data from the proposed study are not publicly available due to privacy or ethical restrictions.

## References

[1] Marjanovic N , Guénézan J , Frat JP , Mimoz O , Thille AW . High‐flow nasal cannula oxygen therapy in acute respiratory failure at emergency departments: a systematic review. Am J Emerg Med. 2020;38(7):1508‐1514. 10.1016/j.ajem.2020.04.091 32389397

[2] Raven MC , Lowe RA , Maselli J , Hsia RY . Comparison of presenting complaint vs discharge diagnosis for identifying “nonemergency” emergency department visits. JAMA. 2013;309(11):1145‐1153. 10.1001/jama.2013.1948 23512061 PMC3711676

[3] Kelly AM , Keijzers G , Klim S , et al. An observational study of dyspnea in emergency departments: the Asia, Australia, and New Zealand Dyspnea in Emergency Departments Study (AANZDEM). Acad Emerg Med. 2017;24(3):328‐336. 10.1111/acem.13118 27743490

[4] Marjanovic N , Flacher A , Drouet L , et al. High‐flow nasal cannula in early emergency department management of acute hypercapnic respiratory failure due to cardiogenic pulmonary edema. Respir Care. 2020;65(9):1241‐1249. 10.4187/respcare.07278 32291308

[5] Ischaki E , Pantazopoulos I , Zakynthinos S . Nasal high flow therapy: a novel treatment rather than a more expensive oxygen device. Eur Respir Rev. 2017;26(145):170028. 10.1183/16000617.0028-2017 28794144 PMC9488954

[6] Mauri T , Turrini C , Eronia N , et al. Physiologic effects of high‐flow nasal cannula in acute hypoxemic respiratory failure. Am J Respir Crit Care Med. 2017;195(9):1207‐1215. 10.1164/rccm.201605-0916OC 27997805

[7] Rochwerg B , Granton D , Wang DX , et al. High flow nasal cannula compared with conventional oxygen therapy for acute hypoxemic respiratory failure: a systematic review and meta‐analysis. Intensive Care Med. 2019;45(5):563‐572. 10.1007/s00134-019-05590-5 30888444

[8] Titler MG . The evidence for evidence‐based practice implementation. In: Hughes RG , ed. Patient Safety and Quality: An Evidence‐Based Handbook for Nurses. Agency for Healthcare Research and Quality; 2008:113‐161. Available from: https://www.ncbi.nlm.nih.gov/pubmed/21328760 21328752

[9] Ruiz VR , Mayer GF , Roux NG , Peralta HA . Use of high‐flow nasal cannula in the emergency department. Eur Respir J. 2016;48:PA357. 10.1183/13993003.congress-2016.PA357

[10] Lenglet H , Sztrymf B , Leroy C , Brun P , Dreyfuss D , Ricard JD . Humidified high flow nasal oxygen during respiratory failure in the emergency department: feasibility and efficacy. Respir Care. 2012;57(11):1873‐1878. 10.4187/respcare.01575 22417844

[11] Bell N , Hutchinson CL , Green TC , Rogan E , Bein KJ , Dinh MM . Randomised control trial of humidified high flow nasal cannulae versus standard oxygen in the emergency department. Emerg Med Australas. 2015;27(6):537‐541. 10.1111/1742-6723.12490 26419650

[12] Makdee O , Monsomboon A , Surabenjawong U , et al. High‐flow nasal cannula versus conventional oxygen therapy in emergency department patients with cardiogenic pulmonary edema: a randomized controlled trial. Ann Emerg Med. 2017;70(4):465‐472. 10.1016/j.annemergmed.2017.03.028 28601264

[13] Jones PG , Kamona S , Doran O , Sawtell F , Wilsher M . Randomized controlled trial of humidified high‐flow nasal oxygen for acute respiratory distress in the emergency department: the HOT‐ER study. Respir Care. 2016;61(3):291‐299. 10.4187/respcare.04252 26577199

[14] Huang CC , Lan HM , Li CJ , et al. Use high‐flow nasal cannula for acute respiratory failure patients in the emergency department: a meta‐analysis study. Emerg Med Int. 2019;2019:2130935. 10.1155/2019/2130935 31737365 PMC6815584

[15] Tinelli V , Cabrini L , Fominskiy E , et al. High flow nasal cannula oxygen vs. conventional oxygen therapy and noninvasive ventilation in emergency department patients: a systematic review and meta‐analysis. J Emerg Med. 2019;57(3):322‐328. 10.1016/j.jemermed.2019.06.033 31421952

[16] Zhu Y , Yin H , Zhang R , Wei J . High‐flow nasal cannula oxygen therapy versus conventional oxygen therapy in patients with acute respiratory failure: a systematic review and meta‐analysis of randomized controlled trials. BMC Pulm Med. 2017;17(1):201. 10.1186/s12890-017-0525-0 29237436 PMC5729290

[17] Liu CW , Cheng SL . Application of high‐flow nasal cannula in COVID‐19: a narrative review. Life (Basel). 2022;12(9):1419. 10.3390/life12091419 36143455 PMC9505799

[18] Heneghan C , Goldacre B , Mahtani KR . Why clinical trial outcomes fail to translate into benefits for patients. Trials. 2017;18(1):122. 10.1186/s13063-017-1870-2 28288676 PMC5348914

[19] Akobeng AK . Understanding randomised controlled trials. Arch Dis Child. 2005;90(8):840‐844. 10.1136/adc.2004.058222 16040885 PMC1720509

[20] Ricard JD , Roca O , Lemiale V , et al. Use of nasal high flow oxygen during acute respiratory failure. Intensive Care Med. 2020;46(12):2238‐2247. 10.1007/s00134-020-06228-7 32901374 PMC7478440

[21] Faraoni D , Schaefer ST . Randomized controlled trials vs. observational studies: why not just live together. BMC Anesthesiol. 2016;16(1):102. 10.1186/s12871-016-0265-3 27769172 PMC5073487

[22] Creswell JW , Creswell JD . Research Design. 5th ed. SAGE Publications; 2018.

[23] Hurtado‐Chong A , Joeris A , Hess D , Blauth M . Improving site selection in clinical studies: a standardised, objective, multistep method and first experience results. BMJ Open. 2017;7(7):e014796. 10.1136/bmjopen-2016-014796 PMC5734283 28706090

[24] Davis P , Lay‐Yee R , Briant R , Ali W , Scott A , Schug S . Adverse events in New Zealand public hospitals I: occurrence and impact. N Z Med J. 2002;115(1167):271. 12552260

[25] ICH Harmonized Tripartite Guideline . Guideline for good clinical practice. J Postgrad Med. 2001;47(1):45‐50. 11590294

[26] Massey University. Code of Responsible Research Conduct. Massey University; 2015. https://www.massey.ac.nz/massey/fms/PolicyGuide/Documents/Research/Code%20of%20Responsible%20Research%20Conduct.pdf?45716EC63691258E8748766DB1EE6E6D

[27] Vandenbroucke JP , von Elm E , Altman DG , et al. Strengthening the reporting of observational studies in epidemiology (STROBE): explanation and elaboration. Epidemiology. 2007;18(6):805‐835. 10.1097/EDE.0b013e3181577511 18049195

[28] Assel M , Sjoberg D , Elders A. Guidelines for reporting of statistics for clinical research in urology. J Urol. 2019;201(3):595‐604. 10.1097/ju.0000000000000001 30633111 PMC6600813

[29] Yang LJS , Chang KWC , Chung KC . Methodologically rigorous clinical research. Plast Reconstr Surg. 2012;129(6):979e‐988e. 10.1097/PRS.0b013e31824eccb7 22634695 PMC3531969

[30] Jakobsen JC , Gluud C , Wetterslev J , Winkel P . When and how should multiple imputation be used for handling missing data in randomised clinical trials—a practical guide with flowcharts. BMC Med Res Methodol. 2017;17(1):162. 10.1186/s12874-017-0442-1 29207961 PMC5717805

[31] Kang H . The prevention and handling of the missing data. Korean J Anesthesiol. 2013;64(5):402‐406. 10.4097/kjae.2013.64.5.402 23741561 PMC3668100

[32] Vetter TR , Mascha EJ . Defining the primary outcomes and justifying secondary outcomes of a study: usually, the fewer, the better. Anesth Analg. 2017;125(2):678‐681. 10.1213/ane.0000000000002224 28682958

[33] Andrade C . Sample size and its importance in research. Indian J Psychol Med. 2020;42(1):102‐103. 10.4103/ijpsym.Ijpsym_504_19 31997873 PMC6970301

[34] Clark‐Carter D . Quantitative Psychological Research: The Complete Student's Companion. Taylor & Francis Group; 2018.

[35] Kim N , Fischer AH , Dyring‐Andersen B , Rosner B , Okoye GA . Research techniques made simple: choosing appropriate statistical methods for clinical research. J Invest Dermatol. 2017;137(10):e173‐e178. 10.1016/j.jid.2017.08.007 28941476

[36] Armstrong RA . When to use the Bonferroni correction. Ophthalmic Physiol Opt. 2014;34(5):502‐508. 10.1111/opo.12131 24697967

